# Peer Review of “Associations Between IT Job Stressors and Anxiety, Depression, and Stress: Cross-Sectional Study”

**DOI:** 10.2196/91383

**Published:** 2026-03-03

**Authors:** Holger Mühlan

**Affiliations:** 1 Universität Greifswald, Greifswald, Germany

**Keywords:** survey, association, occupational health, mental health, stressors, IT, IT professionals, United States, workplace, depression, anxiety, stress, help-seeking, health literacy

*This is a peer review report for “Associations Between IT Job Stressors and Anxiety, Depression, and Stress: Cross-Sectional Study.”*

## Round 1 Review

### General Comments

Although the data basis of this study [1] is not very reliable due to methodological limitations (cross-sectional design, online survey, self-reporting only, self-selection bias), the study does provide some interesting insights. I have the following comments to make.

### Specific Comments

#### Major Comments

What I miss most is a more differentiated discussion of the assumed mediation by mental health literacy (MHL). In my view, it is not necessarily plausible. It would be just as plausible to assume that people with high MHL are more competent in knowing what to do by themselves. In addition, MHL itself can also have a protective function, in that people with high MHL also know what they can do by themselves to protect themselves in the event of a high workload and apply any compensatory measures (eg, relaxation).

#### Minor Comments

1. Some information on the analysis should be added to the abstract.
2. “Anxiety” should also be included in the keywords.
3. With regard to the excluded cases, no cases are mentioned that were excluded due to conspicuous response behavior (eg, monotonous patterns) or too rapid completion (“speeders”). Why?
4. Please add information on how many people in total were initially contacted.
5. A core element of the study is the initial identification of IT-specific stressors. Here, further information on the criteria for the selection of the experts and the methodological procedure for capturing the stressors is essential (interviews? focus group? workshop?).
6. The study just assessed the intention to seek help. Did it also assess whether professional help had been sought in the past 12 months? If not, why not? This would have been very easy to capture and a much more reliable criterion than just intentions.
7. Please add a table with the most important information about the sample.
8. Please also add a table with the frequencies of the individual stressors as well as the distribution of multiple stressors (ie, how often people reported 1 stressor, 2 stressors, etc, up to 12 stressors). This is also interesting, as the effect of multiple stressors appears to be surprisingly small. The type of stressor therefore seems to be more decisive than the frequency/diversity.
9. Table 1 has a different font type, please adjust.
10. Some of the terms in Table 2 are written inconsistently (eg, “p-value”/“P value”).
11. Please add a legend beneath Table 2, explaining the abbreviations of the measures.
12. The discussion basically only addresses why mediation shows no effect with regard to stress, but not why this is also the case with anxiety. Please also address this.
13. Incidentally, the mediation effect for depression should not be overestimated. The effect just tips significance and the size of the indirect effect is rather small relative to the huge direct effect.
14. The assessment of MHL by self-report is not necessarily a limitation per se, as there are both objective and subjective concepts in MHL. The question is rather which version is the more suitable for operationalization for testing your hypotheses.
15. The references are still very inconsistent, eg, some journal titles are abbreviated/others are not; some references lack information (eg, Northwave—Where did “In” appear?) or the year of publication (eg, for Boehm et al [2]). Please check the reference list manually throughout.

## Round 2 Review

The authors addressed all points appropriately and I have not spotted any further issues.

## Abbreviations

MHL: mental health literacy
